# Researcher-Rated “Snapshots” of Stress: Initial Validation of Two Stress Assessment Approaches and Their Relationship to Internalizing Symptoms

**DOI:** 10.1155/da/5522234

**Published:** 2025-10-30

**Authors:** Elli Cole, Gail Corneau, Alessandra R. Grillo, Suzanne Vrshek-Schallhorn

**Affiliations:** University of North Carolina Greensboro, 1400 Spring Garden Street, Greensboro, North Carolina 27412, USA

**Keywords:** anxiety, assessment, depression, perceived stress, stress exposure

## Abstract

Extant questionnaire measures of stress frequently conflate stress exposure and response and can be confounded by factors such as trait neuroticism; by contrast, contextual interviews target stress exposure but require significant resources that are a barrier to swift data collection. We reasoned that it may be possible to use researcher-rated “snapshots” of brief participant-written descriptions of stress to obtain similar independence from trait neuroticism as interviews do, even though such an approach would not provide all the benefits of interview measures. This study evaluates the psychometric properties of this novel stress assessment approach using both researcher and self-report ratings, in part by examining the contribution of these indicators of stress to internalizing subfacets. Adults (*N* = 378) reported on their stress during the COVID-19 pandemic (May–June, 2020) using two measures that covered 11 life domains (~4158 ratings per measure). Inter-rater reliability for researcher ratings of participant stressor descriptions was good, and both self-rated perceived stress and researcher-rated stress had significantly smaller correlations with neuroticism compared to a traditional perceived stress measure, indicating favorable discriminant validity. Both approaches generated acceptable two-factor (interpersonal and noninterpersonal) structures. Interpersonal and noninterpersonal self- and researcher-rated stress were associated with internalizing facets, with some variation. These results provide initial evidence that two novel and rapid methods of measuring stress retain certain appealing properties of life stress interviews (LSIs), for occasions in which interviews are not feasible.

## 1. Introduction

Stress is a potent risk factor for the development of psychopathology, particularly internalizing disorders [1–4] and was elevated during the COVID-19 pandemic [5–7]. Yet, stress presents significant challenges to valid measurement. Many self-report measures conflate stress exposure—what happens in the environment—with the stress response—how the individual perceives the experience and reacts emotionally, cognitively, and biologically [8]. Critically, the stress response can be influenced by factors such as trait neuroticism [8], which is also a strong predictor of internalizing outcomes, thereby confounding prediction when stress is operationalized as the stress response. While researcher-rated interviews more accurately measure stress exposure, they require significant resources and typically cannot be deployed quickly, for example, when real-time, rapid data collection is needed following a natural disaster or pandemic. Furthermore, self-report questionnaire measures of perceived stress tend to assess the construct globally, despite research suggesting the content of the stressor matters (e.g., employment, health, relationships), namely their interpersonal or noninterpersonal nature [9, 10]. This presents an opportunity to explore alternative methods of stress measurement that can be rapidly deployed in the same way self-report measures can, but also may be able to reduce the association with other characteristics, such as neuroticism, and assess multiple domains. Toward these aims, the present study provides a proof of concept of a new method of stress measurement that uses both self-rated perceived stress and researcher ratings of participants' text descriptions (what we refer to as “stress snapshots”) of 11 life domains (modeled after 10 chronic stress domains in the life stress interview [LSI], with the addition of challenge to obtain necessities) during the early COVID-19 pandemic in 378 adults by examining their distributions, inter-rater reliability when applicable, factor structure, and discriminant validity. We then used the resulting composite scores to examine relationships with internalizing symptoms, not only to determine construct validity, but also to assess the impact of the pandemic.

## 2. Assessment of Stress Exposure Versus Responses

Stress is an expansive term used both colloquially and in research to refer to exposure to environmental events and chronic circumstances, and internal reactions to these [8, 11]; it can be assessed using numerous methods. While self-report questionnaires are quick and inexpensive, they capture the participant's appraisal of a stressful event—perceived stress, an aspect of stress responding—less than what occurred—the stress exposure [8]. However, stressor appraisal can be influenced by current symptoms of psychopathology, previous depression diagnoses, and higher trait neuroticism [12, 13]. Furthermore, self-report measures often do not differentiate between types and severities of stressors, which other work suggests matters [9, 10, 14]. Checklists are documented to be vulnerable to inaccurate retrospective reporting and do not address the problem of “intracategory variability,”—lumping together mild and severe manifestations of the same item [15–17]. Similarly, self-report perceived stress measures often ask about stress with broad questions that do not differentiate between the life domains of stress or between interpersonal and noninterpersonal strains [15, 17]. Semi-structured interviews were subsequently developed to target stress exposure (i.e., disregarding emotional reactions) by gathering information about stressor context and impact across multiple life domains of stress (e.g., employment, health, relationships). The ability to probe for context to distinguish minor from severe intracategory events and the use of independent raters to reduce the influence of participant characteristics (personality, psychopathology) on ratings have led researchers to conclude that these are reliable and valid measures of stress exposure [18]. While semi-structured interviews are ideal, they often require significant time and resources, which presents a barrier when the research question necessitates swift data collection and a larger sample size. While participant-generated content can be collected swiftly, it will inevitably still be vulnerable to some of the same deficits of self-report, including a lack of information on timing and comingling of chronic and acute exposure. However, the use of alternative measures may reduce the impact of individual differences in responding (in the same way as interviews do) while retaining the ability to capture data quickly.

Finally, stress response or appraisal can be valuable when properly conceptualized, but there is a need to quantify this separately for different life domains for greater specificity. The most commonly cited measure of perceived stress, Perceived Stress Scale (PSS) [19], assesses feelings of being overwhelmed (e.g.,) without reference to life domains. The present study addressed several gaps by assessing multidomain perceived stress, influenced by methods used in Hammen's UCLA LSI for chronic stress [20], to gather ratings for 11 life domains using a rapidly deployable new approach to measuring stress exposure that retains investigator ratings.

## 3. Stress and Risk for Facets of Internalizing Symptomology

Stress has been consistently linked with internalizing symptomology [1, 2, 10, 14, 21]. Notably, in recent work, interpersonal stress contributed greater unique variance on average to depression than noninterpersonal stress in recent work, but chronic noninterpersonal stress predicted depression onsets with increasing unique variance as socioeconomic status (SES) declined [10]. The COVID-19 pandemic created significant upheaval in numerous areas of daily life (e.g., loss of employment, lack of childcare, separations/loss of relationships, grief), and individuals reported higher rates of perceived stress, depression, and anxiety [6, 22–25]. This novel experience not only represents a period of significant stress, but the stressors may also differ in their relative contributions of interpersonal and noninterpersonal stress to internalizing symptoms (e.g., noninterpersonal stress may become more salient than interpersonal stress or vice-versa). As such, we hypothesized that the new measures of stress would be associated with internalizing symptoms, but we did not have strong expectations about the relative roles of interpersonal or noninterpersonal stress.

## 4. Present Study

The present study probed the measurement properties of two novel approaches to assessment of stress severity in 378 adults during a 5-week period in the early COVID-19 pandemic (including examining distributions, reliability, factor structure, and discriminant validity), then examined the relationship of these new stress measures to internalizing facets, including anxious arousal, anhedonic depression, and general distress. The two approaches comprise self-reported perceived stress ratings on 11 domains (modeled after the LSI, with the addition of difficulty obtaining necessities) and researcher ratings of participants' approximately three-sentence text descriptions (“stress snapshots”) on the same 11 domains. The aim is not to replicate *all* benefits of interviews, but to test a proof of concept whether these novel approaches can provide greater independence from neuroticism compared to self-reported perceived stress, in a similar manner as interviews. We hypothesized that for both assessment approaches (1) interpersonal and noninterpersonal factors would emerge in factor analyses, and (2) each resulting composite would be significantly less correlated with neuroticism compared to a perceived stress measure, suggesting an improvement compared to extant perceived stress measures. Despite hypotheses regarding structure, we chose to employ exploratory factor analysis (EFA) for this initial validation to maximize the novel information derived and not impose our pre-existing expectations. Consistent with prior research that has identified perceived stress as a risk factor for internalizing symptoms, we hypothesized (3) that both participants' perceived stress and researcher-rated stress snapshots would (a) be associated with internalizing facets (anhedonic depression, anxiety, and distress), but also (b) contribute unique variance to internalizing facets over and above the other.

## 5. Methods

### 5.1. Participants

This sample (*N* = 412) was recruited using public social media posts. Participants indicated their informed consent in Qualtrics. Eligible participants were over the age of 18 and proficient in English. We made exclusions (*n* = 34) for inattentive responses on the Infrequent Responses Scale of the Attentive Responding Scale (ARS; [26]) using the recommended cut score of >7.5 out of 24. This resulted in a final sample size of 378 participants, ranging in age from 18 to 88 years old (*M* = 41, SD = 14.09). The sample was predominantly female (87%) with five individuals identifying as either nonbinary or genderqueer (1.3%). The sample was predominantly White (86.7%) with the rest identifying as Latiné (4.4%), African American/Black (3.4%), Asian/Asian American (2.4%), Native American/Pacific Islander (2%), or another race/ethnicity (0.7%). SES was measured using nine income brackets ranging from $14,999 or less to more than $200,000, with higher scores indicating greater income; the median bracket was $75,000–$99,000.

### 5.2. Procedure

Ethical approval for this study was obtained from the institution's review board (20-0404), and all participants provided written informed consent. Data collection began on May 1, 2020, approximately 1.5 months after the World Health Organization declared COVID-19 a pandemic [27], and ended on June 9, 2020. Participants were recruited from the general public through social media posts shared by the principal investigator and a team of research assistants, as well as internal media coverage by the university accessible to the faculty and student body. Additional measures collected beyond the present aims are not reported here. Artificial intelligence was not used in the development, execution, or writing of the present study.

### 5.3. Measures

#### 5.3.1. Internalizing Symptoms

The short form of the Mood and Anxiety Symptom Questionnaire (MASQ-D30; [28]) was used to measure participants' internalizing symptoms. The MASQ-D30 asked participants about the frequency of their experiences in the last week with three subscales, which measured anhedonic depression (e.g., “felt like I was having a lot of fun,” reverse scored), anxious arousal (e.g., “felt dizzy or light-headed”), and distress (“felt dissatisfied with everything”). Participants rated their responses on a 1 (*Not at all*) to 5 (*Extremely*) Likert scale, where higher scores indicated greater intensity of experiences. The items for the anhedonic subscale were reverse-scored. For all scales, up to 20% missing responses were allowed, and items were averaged to generate an item mean. The Cronbach's alpha in this study was acceptable (*α* = 0.86–97).

#### 5.3.2. Two-Part Stress Measure

This measure was developed to measure interpersonal and noninterpersonal stress using elements inspired by the chronic stress portion of the UCLA LSI [20], which captures participants' circumstances on a scale from best possible to worst possible. The present study assessed 11 total life domains: four traditionally believed to be interpersonal (best friend, social circle, romantic partner, and immediate family) and seven believed to be noninterpersonal (residence and neighborhood quality, finances, ability to acquire food and necessary supplies, academics, employment, personal health, and health of one's immediate family). All domains followed the framework of the chronic stress LSI, apart from accessing food and supplies, which we developed for this study, given the difficulties with food and supply shortages during the pandemic. We used two different methods to assess stress on these 11 domains.

##### 5.3.2.1. Self-Rated Perceived Stress

First, participants self-rated the severity of their perceived stress, meaning the degree to which they *felt* the pandemic impacted their life experiences across the 11 domains using a 9-point Likert scale ranging from −4 (*Most negatively impacted*) to +4 (*Most positively impacted*), where 0 indicates no impact; participants could also select “Not applicable” (e.g., the academic domain if not enrolled in school) which was treated as missing data. One rating per composite was permitted to be missing. Items were reverse-scored so that higher scores reflected more strain, and were converted into *Z*-scores to equally weight the domains in the composite scores, then averaged to generate interpersonal and noninterpersonal self-rated perceived stress composite scores, with higher scores indicating higher perceived stress. Based on the results of the factor analysis described below, composite scores were created to reflect the factors that emerged (i.e., interpersonal and noninterpersonal stress composites), consistent with prior stress research [10], to reduce multiple testing, and to avoid possible multicollinearity among predictors. See Supporting Information 1: Part 1 for the full measure.

##### 5.3.2.2. Researcher-Rated Stress Snapshots

After making perceived ratings of impact, participants were instructed to write brief three-sentence statements that factually described, using objective details, how each of the 11 life domains had changed for better or for worse due to the pandemic. Participants could spontaneously provide content, but example content was provided (e.g., your description could include: how close and trusting your relationship is, how often you have contact, etc.). Participants were asked to omit how they *felt* about the events to limit the effects of emotional responses on researchers' ratings of these statements and maximize researcher objectivity. Participants answered questions indicating they understood these instructions. These text responses were then rated by two independent raters using the same −4 (*Most negatively impacted*) to +4 (*Most positively impacted*) scale used by the participants when making their perceived stress ratings. Raters were trained postbaccalaureate and advanced undergraduate research assistants who were unaware of participants' subjective ratings of stress in each domain. Raters' training included readings about stress exposure, a didactic training with the principal investigator, a “quiz” on a practice battery of internal “gold standard” items with feedback, and a clearance “test” requiring 80% match accuracy to internal “gold standards.” All participant responses were rated twice. If ratings were sufficiently matching (within two points on the 9-point scale), scores of the two raters were averaged to create a single value; for remaining responses, a third rater (deemed by the research team to have high rating proficiency) rerated the free response, and this was used in place of the average score (811 responses or 18.62% were rerated by the 3rd rater). Furthermore, all raters flagged free responses for three types of patterns that did not adhere to the instructions; these included responses that contained excessive emotion that could potentially bias a rater (57 or 1.31% identified by at least 1 rater), instances when the response addressed the wrong domain (2 or 0.05% identified by at least 1 rater), and responses that lacked sufficient content (883 or 20.27% identified by at least 1 rater). The individual domain responses that were flagged by one or more raters were treated as missing data and did not contribute to the composites, but the participant's other data were used unless the participant exceeded our limit for missing data on a composite (>1 rating per composite), and then the composite score was excluded from analyses. Intraclass coefficient values (ICCs) were calculated to estimate inter-rater agreement for these dimensional indicators of stress exposure; ICCs indicated strong agreement between raters for all 11 domains (0.91–0.97). Researcher ratings were reverse-scored so higher scores indicated greater stress exposure to facilitate interpretation of effect sizes predicting adverse mood outcomes, and were converted to *Z*-scores to ensure equivalent weights for each scale in the composites. These *Z*-scores were averaged to create composite scores aligning with the results of factor analysis—interpersonal and noninterpersonal scores. See Supporting Information 2: Part 2 for the full measure.

### 5.4. Neuroticism

Neuroticism was measured using the 10-item International Personality Item Pool representation of the NEO-PI-R neuroticism scale [29]. Participants rated the items (e.g., “am often down in the dumps”) using a scale ranging from 1 (*Very inaccurate*) to 5 (*Very accurate*), where higher scores indicate greater neuroticism. Five items were reverse-scored. We averaged items, allowing 20% missing responses, yielding an item-mean (*α* = 0.88).

### 5.5. Perceived Stress

Perceived stress was measured using Cohen's PSS [19]. The PSS consists of 14 items assessing the participant's levels of stress in the last month (e.g., *In the last month*, *how often have you felt nervous and “stressed”?*) using a five-point Likert scale ranging from 0 (*Never*) to 4 (*Very often*), where higher scores represent greater perceived stress. Seven items were reverse-scored, and items were averaged, allowing for 20% missing, yielding an item-mean (*α* = 0.89).

### 5.6. Analytic Plan

**Figure 1:**
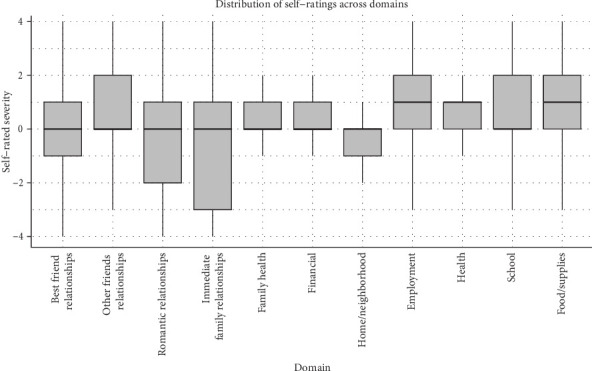
Boxplots of self-rated stress across 11 interpersonal domains. Note: Boxplots depict transformed scores where higher scores indicate greater stress, scores of 0 reflect no change, and negative scores reflect a reported reduction in strain.

**Figure 2:**
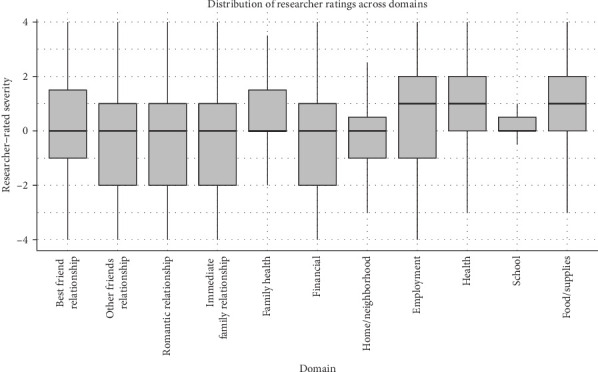
Boxplots of researcher-rated stress across 11 noninterpersonal domains. Note: Boxplots depict transformed scores where higher scores indicate greater stress, scores of 0 reflect no change, and negative scores reflect a reported reduction in strain.

All analyses were conducted using SPSS (Version 26.0) and Mplus (Version 8.8). Descriptive statistics and correlations were calculated among the variables of interest, and the three dependent variables, anhedonic depression, anxious arousal, and distress, were examined for normality. Boxplots were used to provide a visual depiction of the data (Figures 1 and 2). Histograms and descriptive statistics were used to assess the distribution of the individual domains. Prior research using chronic LSIs suggests a normal distribution would be expected [10].

To test Hypothesis 1, whether there were meaningful factors for stress domains, EFAs were used for both self- and researcher-rated measures. Although theory suggested that interpersonal and noninterpersonal factors would emerge, EFA (rather than confirmatory factor analysis) was chosen to provide maximum information. 2 These results were then used to inform the creation of composite variables. To test Hypothesis 2, whether the composite scales of the new stress measures demonstrated adequate discriminant validity when compared to trait neuroticism, Pearson zero-order correlations between the two new measures and neuroticism, as well as between Cohen's PSS and neuroticism, were calculated. The *r*-values were then compared using a *Z*-test of dependent correlations [30].

To test Hypothesis 3a, whether self- and researcher-rated measures of stress would be associated with internalizing facets, we first ran Pearson zero-order correlations of self- and researcher-rated composite scores with internalizing facets. Next, for Hypothesis 3b, to examine whether self- and researcher-rated composites contributed unique variance to internalizing outcomes, we conducted three separate regression analyses predicting each internalizing facet in turn. This was repeated with the noninterpersonal composite scores for self- and researcher-rated stress. The *N* for each analysis varied slightly depending on the amount of missing data for each predictor and outcome. Gender, race/ethnicity, and SES were included as covariates, given their respective associations with depression [1, 2]. Two dummy-coded variables were used to separate the three categories of male, female, or gender nonbinary, leaving male as the reference category. Race and ethnicity responses were coded as either not historically marginalized race or ethnicity (0) or historically marginalized race or ethnicity (1). To probe these latter multiple regression models in an exploratory post hoc manner, using Mplus (8.8), we compared the magnitude of effects between self-rated perceived stress and researcher-rated stress snapshots by using deviance tests between constrained and unconstrained models.

## 6. Results

### 6.1. Structural Analyses

#### 6.1.1. Self-Rated Perceived Stress

Multiple EFAs were conducted with both oblique rotations; correlations between factors were expected. A principal axis factor extraction with a promax rotation was used in the final model, given optimal minimization of cross-loadings between factors. The results indicated that a two-factor structure fit the data best, accounting for ~42% of the variance. The following domains loaded onto one factor: relationships with best friends, broader social friend circle, immediate family, romantic partner, along with family health and (perhaps surprisingly) education, with all factor loadings greater than or equal to 0.30 (Table 1). All other items (neighborhood, financial, employment, personal health, food/supplies) loaded onto the second factor with factor loadings greater than or equal to 0.30. The correlation between the two factors was 0.48.

#### 6.1.2. Researcher-Rated Stress

Similar to perceived stress ratings, researcher-rated stress had a two-factor structure. Principal axis factor extraction with a promax rotation was also used, with the model accounting for ~31% of the variance. The structure suggested the presence of interpersonal and noninterpersonal factors and was similar to the structure of the self-rated measure, with the exception of education and family health. Although family health and education loaded more strongly onto the *interpersonal* factor in the perceived stress EFA, these components loaded more strongly onto the *noninterpersonal* factor in the researcher-rated EFA (Table 2). The two factors' correlation was 0.48. To maintain consistency across self- and researcher-rated measures when forming composite scores for the remaining analyses, in both sets of composite scores, family health was included in the noninterpersonal domains in accordance with stress literature [31]. Furthermore, given that academics did not consistently load onto a single factor and had some evidence of cross-loading, it was included with the other noninterpersonal domains. 1 For each approach, self-rated perceived stress and researcher-rated stress snapshots, there were two domains classified as interpersonal (relationships with best friend, other friends, romantic partner, family, and family health) and noninterpersonal (neighborhood quality, finances, employment, personal health, academics, and access to food and necessities). Results predominantly supported Hypothesis 1.

### 6.2. Descriptive Statistics and Dependent Correlations

Significant, moderate, positive zero-order correlations were found between perceived interpersonal and noninterpersonal stress as well as researcher-rated interpersonal and noninterpersonal stress measures (*r* = 0.21–0.26, *p*'s all <0.05), as anticipated (Table 3). Despite asking about the same domains and content, correlations between the self- and researcher-rated measures corresponding to interpersonal and noninterpersonal domains were moderate (*r* = 0.45–0.46, *p*'s all <0.05). Inspection of the distribution of individual domains (e.g., family health, finances) demonstrated use of the full range, indicating that participants endorsed experiences spanning from the most negative change possible to the most positive change possible (Figures 1 and 2). The distribution of self- and researcher-rated stress was as expected, with a relatively normal distribution, where the range of skewness fell within +/−1.2 and kurtosis was within +/−3. Histograms indicated a normal distribution across domains in general, apart from academics, which showed restricted variability, potentially due to low rates of students within this sample.

Results supported Hypothesis 2, with both self-rated and researcher-rated measures discriminating between overall perceived stress and neuroticism similarly. *Z*-tests of dependent correlations tested differences between correlations among perceived stress, neuroticism, and the four self- and researcher-rated stress composites. In each case, the correlation between neuroticism and perceived stress (*r* = 0.62, *p* < 0.001) was significantly larger than the respective correlation between the new index and neuroticism, self-rated perceived interpersonal stress (*r* = 0.19, *p* < 0.001; *z* = 7.55, *n* = 372, *p* < 0.001), self-rated perceived noninterpersonal stress (*r* = 0.20, *p* < 0.001; *z* = 8.65, *n* = 372, *p* < 0.001), researcher-rated interpersonal stress (*r* = 0.17, *p*=0.001; *z* = 7.95, *n* = 357, *p* < 0.001), and researcher-rated noninterpersonal stress (*r* = 0.30, *p* < 0.001; *z* = 5.88, *n* = 317, *p* < 0.001). Notably, correlations of self-rated perceived stress with neuroticism were within a descriptively similar range as those of researcher-rated stress snapshots. Results supported Hypothesis 2.

### 6.3. Self- and Researcher-Rated Stress Predicting Internalizing Facets

There was general support for Hypothesis 3a; zero-order correlations demonstrated significant relationships between the four composite measures of self- and researcher-rated stress and anhedonic depression and general distress (*r* = 0.16–0.30, *p* < 0.05; Table 3), and all but researcher-rated noninterpersonal stress were associated with anxious arousal (*r* = 0.10, *p*=0.07). These results provide evidence of the construct validity of both new methods of stress assessment.

Hypothesis 3b, that both self- and researcher-ratings would contribute unique variance to all internalizing facets, had mixed results (Tables 4 and 5). When self- and researcher-rated *interpersonal* stress were simultaneously entered into the model, self-rated perceived stress significantly predicted anxious arousal (*β* = 0.16, *p*=0.009), general distress (*β* = 0.18, *p*=0.002, and anhedonic depression (*β* = 0.13, *p*=0.027). However, only researcher-rated interpersonal stress contributed significant unique variance to general distress (*β* = 0.13, *p*=0.029). In contrast, researcher-rated *noninterpersonal* stress was a significant predictor of all three facets (*β* = 0.13–0.17, *p* < 0.05), while self-rated perceived noninterpersonal stress was a significant predictor of only general distress (*β* = 0.16, *p*=0.008) but not anhedonic depression or anxious arousal. Deviance tests determined differences in the magnitude of effects between predictors, and only self-rated interpersonal stress contributed significantly greater unique variance than researcher-ratings when predicting anxious arousal, *χ*^2^(1, *N* = 292) = 4.78, *p*=0.029. Because both approaches tended to contribute unique variance and their unique contributions did not generally differ, the results support using either or both self- and researcher-ratings of stress.

## 7. Discussion

This study provides an initial proof of concept and preliminary support for two new stress assessment methods that utilize self-reported ratings of perceived stress and researcher ratings of participant-generated text descriptions of their circumstances (“stress snapshots”) across multiple life domains in the early COVID-19 pandemic. The results show that these measures capture a full range of negative to positive conditions, have good inter-rater reliability when applicable, reduce correlations with neuroticism compared to a traditional perceived stress measure (a primary goal of this effort), and are feasible when data must be collected swiftly. Furthermore, the structure of the measure roughly approximated the interpersonal and noninterpersonal domains traditionally used to create composite scores in the chronic LSI literature, and these composites were associated with internalizing symptoms in almost all cases. To varying degrees noted below, both methods of measurement (self- and researcher-ratings) contributed unique variance to each facet of internalizing, with neither significantly outperforming the other, with the exception of self-rated perceived interpersonal stress, which predicted significantly more unique variance in anxious arousal compared to researcher-rated stress. Taken together, these results support the value of both methods of measuring stress; while they cannot provide all the benefits of interview measures, they provide certain key benefits.

### 7.1. Structure of Self- and Researcher-Rated Stress Measurement

The results of two EFAs demonstrated that overall, these two measures generally aligned with the interpersonal and noninterpersonal domains often formed from the chronic stress LSI [20], with slight differences across the self- and researcher-rated approaches necessitating judgment to form the final factors. Despite some variability in the factor loadings across the self- and researcher-rated approaches, the factor loadings of the two domains generally aligned with the structure of the LSI. Only family health and academics did not cleanly fall into each category, with family health cross-loading onto both factors in the self-rated perceived stress measure, and academics loading onto the interpersonal domain for self-rated perceived stress and the noninterpersonal domain for researcher ratings; the domains generally aligned with the structure of the LSI. It is possible that the inconsistency in loadings seen in the academic domain was due to very few participants in the sample (average age ~42 years) actively engaged in academics, and a compression of range for this domain. The cross-loading of family health onto both domains could be attributed to the COVID-19 pandemic, which was characterized by significant stress about health broadly [5], coupled with increased separation from loved ones living outside the home (or even quarantining within the home). Furthermore, acute family health concerns that were not noninterpersonally specific (helping with doctors' appointments, medication), may have also impacted participants' quality and quantity of close, interpersonal relationships—the very definition of interpersonal stress.

### 7.2. Self-Rated Perceived Stress

This study offers initial evidence for a self-rated measure of perceived stress that provides several benefits beyond those available in existing perceived stress measures (e.g., PSS, State–Trait Stress Inventory, Daily Hassles Scale). This perceived stress measure does not replace, but complements researcher-rated tools of stress exposure (e.g., LSI) by asking participants to rate how they *feel* they were impacted (on a broad spectrum from positively to negatively), capturing not only their perception of each stressor but whether there were, in fact, positive changes in life domains. Given that this is a perceived stress measure and as such might be expected to be more related to neuroticism, the magnitude of improved discriminant validity was surprising to us. It was significantly less correlated with neuroticism than a traditional perceived stress measure. It is possible that asking participants to direct their attention to specific life domains as opposed to rating overall perceived stress (such as the extent to which they generally “feel overwhelmed”) reduced the association with neuroticism. As such, we conclude this perceived stress measure offers both greater granularity for life domains and reduced correlation with neuroticism, and could enhance the measurement of perceived stress by capturing a greater range of experiences than other widely used perceived stress measures.

Furthermore, both interpersonal and noninterpersonal self-rated perceived stress were significantly associated with internalizing facets in zero-order correlations, supporting Hypothesis 3a. Moreover, both interpersonal and noninterpersonal self-rated perceived stress contributed unique variance (accounting for respective researcher-rated stress exposure snapshots) to general distress. By contrast, neither interpersonal nor noninterpersonal self-rated perceived stress composite contributed significant unique variance to anhedonic depression, above and beyond the researcher-ratings; while this was unexpected, because the difference in these contributions was not significantly different, we do not make inferences about this finding. However, this finding should be monitored in replication efforts. Interestingly, interpersonal self-rated perceived stress contributed significantly more variance to anxious arousal than did the researcher-ratings, but noninterpersonal self-rated perceived stress did not contribute significant unique variance over and above researcher-ratings. Overall, the results are generally consistent with expectations, where interpersonal and noninterpersonal perceived stress were associated with internalizing facets with some variation, which further demonstrates evidence of construct validity.

### 7.3. Researcher-Rated Measure of Stress

Like the self-rated perceived stress measure, this study provided initial evidence of validity for independent ratings of the brief narrative “snapshots” provided by participants across 11 domains. Overall, this measure demonstrated strong inter-rater reliability, and critically, like the self-rated perceived stress measure, the researcher-rated stress snapshots had significantly smaller correlations with neuroticism than did a traditional perceived stress measure (PSS). Unlike traditional self-report measures (e.g., PSS, Daily Hassles Scale), this researcher-rated measure asked participants to describe their circumstances rather than their feelings about them, and responses were excluded from analyses if the participant included emotional content. These procedures, combined with the use of independent raters, likely contributed to the reduced association between the measure and neuroticism when compared to a traditional perceived stress measure. We do not definitively conclude that this measure captures stress exposure, first, because we were not able to collect contextual interviews (the gold-standard approach to measuring stress exposure) for comparison. Second, because participants select what aspects of each life domain to report on, rather than interviewers, our approach does not directly replicate systematically probing the same aspects of each life domain across participants. Furthermore, while the questions inquired about how the pandemic affected each area of their lives, which did have a relatively specific start date (e.g., start of lockdowns), we did not ask for dates of onset or attempt to differentiate between chronic and episodic (acute event) stressors. However, instructions not to provide their feelings about the impact, employing independent raters, instructing raters to ignore minor statements about emotional responses in their ratings, and excluding responses that contained substantial emotion counter to instructions seemed to limit the association with neuroticism. While this measure was collected during a pandemic, many of the stressors, even those such as lack of access to resources and food insecurity, may be pertinent and even a growing concern regardless of a global pandemic. Future work could examine the feasibility and validity of collecting open-ended text-based responses to specific aspects of each life domain in much the same way that the LSI does so, and assess stress outside of the context of a pandemic. The researcher-rated interpersonal and noninterpersonal composites all had significant zero-order associations with the facets of internalizing symptoms, with the exception of the interpersonal composite's association with anxious arousal. Furthermore, while researcher-rated interpersonal stress did not contribute significant unique variance (beyond the self-rated composite and covariates) to anhedonic depression or anxious arousal, it did contribute significant unique variance to general distress. Researcher-rated noninterpersonal stress, however, contributed significant unique variance to all three internalizing facets, and was the *only* significant unique predictor for both anhedonic depression and anxious arousal. These results provide preliminary evidence that the use of researcher ratings adds incremental variance in associations with internalizing symptoms. Overall, these results indicate that these researcher ratings, which require significantly fewer resources than interviews, show evidence of preliminary validity and can enrich stress measurement when interviews are not feasible.

### 7.4. Comparison of the Two Approaches to Stress Assessment

Given that results generally showed that each approach contributed significant unique variance to one or more facets of internalizing, and with one exception, deviance tests showed no significant differences between their unique contributions to the facets, we conclude that both approaches are likely to be useful to study the effects of stress on internalizing symptoms. Both self- and researcher ratings were less significantly associated with neuroticism compared to a traditional measure of stress, which is advantageous when researchers wish to study constructs such as stress and internalizing outcomes, where neuroticism can be a confounding variable. Furthermore, despite asking about stressful experiences across the same domains, the correlations between self- and researcher-ratings were moderate (*r* = 0.45–0.46, *p*'s  < 0.05), providing further evidence that each measure may be capturing both perceptions of stress and exposure. The two approaches presented in this study can be used conjointly (but not equivalently) to provide complementary analyses of perceived stress and an approximation of stress exposure, or could be used individually when the research question prioritizes one construct or the other.

### 7.5. Future Directions

These results provide initial validation of new measures that can be used in instances where rapid data collection about life stress is necessary. No approach to stress measurement is without drawbacks, including the present approach, and future work could build on this initial effort. First, the researcher-rated snapshots were only three sentences, which is relatively brief. Requiring a word count before the survey system accepts the response could address this issue and may reduce the number of responses that would need to be excluded later for insufficient substantive content. Second, unlike interviews, raters could not query and probe the responses to gather context surrounding the stress exposure described, meaning raters were reliant on the participant to provide sufficient detail for each domain. These drawbacks could be addressed by asking an additional series of open-ended questions, more consistent with a chronic stress interview in questionnaire format, to gather contextual information. Furthermore, this study did not directly compare these methods to contextual LSIs, which is a necessary next step to substantiate their validity and the extent to which they are assessing stress exposure. Therefore, the use of a word count to encourage detailed reports, more specific questions about context built into the questionnaire to increase the similarity of content coverage and depth across participants, and comparison to LSIs are questions for future research. While the present research is nonclinical in nature, we believe that further development of straightforward tools for measuring stress exposure (rather than perceived stress) could help clinicians evaluate where client sources of chronic strain come from, relative to normative data, to streamline problem-solving efforts and coping.

## 8. Limitations

Further, this study was not without limitations. First, because of the need to rapidly deploy the study in the pandemic, we did not collect contextual interviews for comparison. Second, participants were relatively homogenous (excluding age) with high rates of middle- or high-SES, White, female participants. Given that the most serious effects, both psychological and economic, of the pandemic were unjustly borne by people of color and lower-income communities [32–34], our results may have differed in a more diverse sample.

## 9. Conclusion

Questionnaire measures of stress are efficient but suffer from drawbacks, including conflating stress exposures with stress response, while stress interviews tease apart stress exposure from response, but require significant resources, impeding rapid deployment. This study provides preliminary evidence for two new stress assessment tools that offer a middle ground, which were significantly less correlated with neuroticism than common perceived stress measures, and that each contributed significant unique variance in association with internalizing facets. The first, a self-reported perceived measure of negative impact, advances perceived stress measures by obtaining ratings specific to multiple life domains. The second, researcher ratings of participants' text-based “stress snapshots,” is not a replacement for interviews, but may provide some of the beneficial properties of interviews, including more objectively rated information compared to a self-report questionnaire, while also being feasible when swift data collection is needed. Together, these questionnaires may be valuable additions to the field of stress research and assessment.

## Figures and Tables

**Table 1:** Exploratory factor analysis factor loadings for self-rated perceived stress.

Items	Interpersonal	Noninterpersonal
Best friend relationships	**0.77**	−0.10
Other friend relationships	**0.69**	−0.04
Romantic relationships	**0.45**	0.06
Immediate family relationships	**0.53**	0.08
Family health	**0.30**	0.24
Academics	**0.31**	0.11
Neighborhood	−0.01	**0.47**
Financial	−0.14	**0.64**
Employment	0.10	**0.37**
My health	0.15	**0.43**
Food/supplies	0.05	**0.56**

*Note:* Bolded values indicate factor loadings greater than 0.30.

**Table 2:** Exploratory factor analysis factor loadings for researcher-rated stress.

Items	Interpersonal	Noninterpersonal
Best friend relationships	**0.39**	0.01
Other friend relationships	**0.48**	−0.10
Romantic relationships	**0.38**	0.07
Immediate family relationships	**0.39**	0.04
Family health	0.10	**0.30**
Academics	−0.06	0.27
Neighborhood	−0.02	**0.50**
Financial	−0.08	**0.51**
Employment	0.04	**0.36**
My health	0.07	**0.40**
Food/supplies	0.07	**0.31**

*Note:* Bolded values indicate factor loadings greater than 0.30.

**Table 3:** Correlations among self- and researcher-rated stress and internalizing facets.

Variable name	1	2	3	4	5	6	7	8
1. SES^a^	—							
2. Self-rated interpersonal stress	−0.006	—						
3. Self-rated noninterpersonal stress	−0.15*⁣*^*∗∗*^	0.21*⁣*^*∗∗*^	—					
4. Researcher-rated interpersonal stress	−0.10	0.45*⁣*^*∗∗*^	0.18*⁣*^*∗∗*^	—				
5. Researcher-rated noninterpersonal Stress	−0.26*⁣*^*∗∗*^	0.16*⁣*^*∗∗*^	0.46*⁣*^*∗∗*^	0.26*⁣*^*∗∗*^	—			
6. General distress	−0.26*⁣*^*∗∗*^	0.23*⁣*^*∗∗*^	0.28*⁣*^*∗∗*^	0.24*⁣*^*∗∗*^	0.30*⁣*^*∗∗*^	—		
7. Anxious arousal	−0.17*⁣*^*∗∗*^	0.15*⁣*^*∗∗*^	0.17*⁣*^*∗∗*^	0.10	0.21*⁣*^*∗∗*^	0.49*⁣*^*∗∗*^	—	
8. Anhedonic depression	−0.15*⁣*^*∗∗*^	0.16*⁣*^*∗∗*^	0.21*⁣*^*∗∗*^	0.18*⁣*^*∗*^	0.26*⁣*^*∗∗*^	0.60*⁣*^*∗∗*^	0.29*⁣*^*∗∗*^	—
*M*	7.12	−0.27	0.31	−0.07	0.22	2.44	1.54	3.55
SD	1.87	1.48	0.92	1.22	0.93	0.94	0.54	0.85

^a^SES 7 = $75,000–$99,000.

*⁣* ^*∗*^ *p* < 0.05.

*⁣* ^*∗∗*^ *p* < 0.01.

**Table 4:** Self-rated and researcher-rated interpersonal stress predicting internalizing symptoms.

Interpersonal stress									
Variable name	Anhedonic depression			Anxious arousal			Distress		
	*B* (SE)	*β*	*p*	*B* (SE)	*β*	*p*	*B* (SE)	*β*	*p*
Female	0.27 (0.14)	0.11	0.052	0.12 (0.09)	0.08	0.186	0.30 (0.15)	0.11	**0**.**044**
Gender fluid	0.27 (0.30)	0.05	0.364	−0.03 (0.19)	−0.01	0.873	0.02 (0.32)	0.004	0.944
Race/ethnicity	−0.06 (0.13)	-0.02	0.670	−0.07 (0.08)	−0.05	0.382	>0.001 (0.14)	>0.001	0.997
SES	−0.05 (0.02)	-0.11	**0.040**	−0.04 (0.02)	−0.16	**0.003**	−0.11 (0.03)	−0.22	**<0.001**
Self-rated	0.10 (0.07)	0.08	0.158	0.11 (0.05)	0.14	**0.023**	0.22 (0.08)	0.16	**0.004**
Researcher-rated	0.10 (0.07)	0.13	**0.027**	0.01 (0.03)	0.02	0.686	0.12 (0.04)	0.16	**0.004**
*R* ^2^	0.06		**0.001**	0.06		**0**.**004**	0.14		**<0.001**
*N*	359			352			359		

*Note:* Bolded values are significant at *p* < 0.05.

**Table 5:** Self-rated and researcher-rated noninterpersonal stress predicting internalizing symptoms.

Noninterpersonal stress									
Variable name	Anhedonic depression			Anxious arousal			Distress		
	*B* (SE)	*β*	*p*	*B* (SE)	*β*	*p*	*B* (SE)	*β*	*p*
Female	0.23 (0.14)	0.10	0.107	0.10 (0.09)	0.06	0.307	0.25 (0.16)	0.09	0.114
Gender fluid	0.21 (0.31)	0.04	0.497	−0.08 (0.20)	−0.03	0.683	−0.04 (0.34)	−0.01	0.913
Race/ethnicity	−0.08 (0.14)	−0.03	0.574	−0.06 (0.09)	−0.04	0.470	−0.02 (0.15)	−0.01	0.900
SES	−0.02 (0.03)	-0.05	0.340	−0.03 (0.02)	−0.12	**0.046**	−0.10 (0.03)	−0.19	**<0.001**
Self-rated	0.15 (0.09)	0.10	0.106	0.07 (0.06)	0.07	0.233	0.27 (0.10)	0.16	**0 .008**
Researcher-rated	0.17 (0.06)	0.19	**0.003**	0.08 (0.04)	0.13	**0.042**	0.19 (0.06)	0.18	**0.003**
*R* ^2^	0.08		**<0.001**	0.06		**0.003**	0.14		**<0.001**
*N*	326			319			326		

*Note:* Bolded values are significant at *p* < 0.05.

## Data Availability

The participants provided written consent for their deidentified data to be shared publicly, but did not provide consent for their qualitative data to be released or directly quoted due to possible identifying information being revealed. Given the sensitive nature of the research, data will only be made available upon request.

## Endnotes

^1^At the request of an anonymous reviewer, we also provide results of a confirmatory factor analysis (CFA) testing the fit of the data to two theoretical factors—interpersonal (romantic, best friend, family, and other social circle relationships) and noninterpersonal chronic stress (neighborhood, school, employment, financial, food/supplies, personal health, and family health). The researcher-rated stress measures demonstrated good model fit with interpersonal and noninterpersonal factors (*χ*^2^(26) = 32.07, *p*=0.191, CFI = 0.914, RMSEA = 0.044, SRMR = 0.064). The self-rated perceived stress measures approached acceptable model fit (*χ*^2^(39) = 63.72, *p*=0.007, CFI = 0.869, RMSEA = 0.074, SRMR = 0.073) with two factors—interpersonal and noninterpersonal factors. This model for self-rated perceived stress included correlated error variances between food/supplies and finances, best friend and other friend relationships, school and employment, and neighborhood and personal health, based on modification indices and theory supporting relationships between these variables.

^2^To ensure that results were robust to this decision, all analyses were reconducted with the academic area removed. The overall pattern of significance in regression results remained the same, with very minor variations in beta values. Results available from the first author upon request.

## Supporting Information

Supporting Information 1 Self-Reported Stress Questionnaire. The questionnaire used in this study to assess participants' ratings across the 11 domains, and the prompt used for participant-generated descriptions of life events for each of the 11 domains.

Supporting Information 2 TREND (Transparent Reporting of Evaluations with Nonrandomized Designs) Checklist. In accordance with Depression and Anxiety's policies and in support of data transparency and accountability, we have provided a document describing how our study meets criteria for the TREND checklist.
