# Supplementary material for: Researcher-Rated “Snapshots” of Stress: Initial Validation of Two Stress Assessment Approaches and Their Relationship to Internalizing Symptoms
Source: Depress Anxiety. 2025 Oct 30;2025:5522234. doi: 10.1155/da/5522234 (PMC12591814; doi:10.1155/da/5522234)
Supplement: Supporting Information 1 — Self-Reported Stress Questionnaire. The questionnaire used in this study to assess participants' ratings across the 11 domains, and the prompt used for participant-generated descriptions of life events for each of the 11 domains. [file 5522234.f1.docx]

**Supplemental Materials**

**S1. Self-Rated Perceived Stress Questionnaire**

To what extent does it FEEL to you that your life been negatively impacted in each of the following areas, on a scale from -4 to +4, where -4 is the most NEGATIVELY impacted as possible by the pandemic, 0 is not impacted either positively or negatively and +4 is the most POSITIVELY impacted as possible by the pandemic?

-4 The most NEGATIVELY impacted as someone could be by the pandemic

-3

-2

-1

0 Not impacted – neither positively or negatively by pandemic

+1

+2

+3

+4 The most POSITIVELY impacted as someone could be by the pandemic

* options include Not Applicable

1. Quality of relationship with a best friend (or does not apply)
2. Quality/quantity of relationships with friends in your social circle other than a best friend (or does not apply)
3. Romantic relationship (or does not apply)
4. Quality of relationships with immediate family members (or does not apply)
5. Residence/neighborhood quality (stability, safety, and noise)
6. My household’s financial resources
7. My ability to acquire the supplies and food I need for my household (time and availability in stores, not your financial resources)
8. My schooling (or does not apply)
9. My employment (or does not apply)
10. My own health
11. The health of my immediate family members (or does not apply if no immediate family)

**S2. Researcher-Rated Stress “Snapshots” Questionnaire**

Next, we would like you to write some short, factual statements that describe how your life in each area has changed since the start of the COVID19 pandemic. This will help us understand your objective circumstances.

*Instructions:*

We want you to do this in a specific way*.*

- Please do not include how you are *feeling* about things, and how you are doing *emotionally*.
- Please tell us about objective details that describe your life circumstances, including changes resulting from the pandemic.
  - Example: I used to see my best friend daily, but I have not seen him in person for six weeks.
- It would be very helpful if you would write about three sentences per area.

*Comprehension Check:*

To check that you understand the directions, please answer the true/false questions:

- I should write about objective details and not how I am feeling about things
- I should write three sentences
- I should think about the objective changes to my life in each area before and after the COVID19 outbreak.

[Feedback provided that all should be true]

1. Quality of relationship with a best friend (or does not apply). Your description could include:

- how often you have contact,
- how close and trusting your relationship is, and
- how often you argue / how well you resolve conflicts.

[Open-ended response]

1. Quality and quantity of relationships with friends in your social circle other than a best friend (or does not apply if no social circle). Your description could include:

- how many close friends you have,
- how often you can interaction with them,
- how close and trusting your relationships with them are, and
- how often you argue / how well you resolve conflicts.

[Open-ended response]

1. Romantic relationship (or does not apply) Your description could include

- how often you have contact,
- how close and trusting your relationship is, and
- how often you argue / how well you resolve conflicts,
- whether you are the victim of domestic violence.

[Open-ended response]

1. Quality of relationships with immediate family members. Your description could include:

- how often you have contact,
- how close and trusting your relationships are, and
- how often you argue / how well you resolve conflicts.

[Open-ended response]

1. Residence and neighborhood stability, safety, and noise. Your description could include:

- what type of residence you live in (house, apartment, homeless, etc),
- whether you have privacy and space in your residence,
- how much your neighborhood experiences people moving in and out frequently,
- how much crime your immediate neighborhood experiences, and
- whether noise interferes with your ability to sleep and work/study.

[Open-ended response]

1. My household’s financial resources, Your description could include:

- to what extent you have enough money to pay for your household’s housing, utilities, food, clothing, transportation, and education,
- whether you can afford to get carry out food (takeout),
- whether you can afford “extras” with “disposal income” for leisure activities,
- to what extent you have debt, and
- to what extent you have emergency funds in savings

[Open-ended response]

1. My ability to acquire the supplies and food I need for my household (time and availability in stores, *not your financial resources*). Your description could include:

- availability of time to go shopping,
- access to transportation to the store,
- changes in when you need to shop and how long it takes you to shop,
- the availability of fresh foods within your typical price-range & quantity in stores,
- the availability of household goods like toilet paper and cleaning supplies in your typical price-range & quantity in stores.

[Open-ended response]

1. My schooling (or does not apply) Your description could include:

- the quality of relationships with your instructors,
- your access to the instructors,
- your ability to attend classes / access lectures,
- the quality of your own study skills,
- the extent to which you have sufficient time to devote to your studies,
- whether your studies are applicable to your future career,
- how you are generally performing in your studies (e.g., grades, awards, academic probation).

[Open-ended response]

1. My employment (or does not apply) Your description could include:

- whether you have the right amount of work hours (or too much, or too little),
- whether your workplace is safe including access to protective equipment if needed,
- whether your work provides opportunities for advancement,
- whether your bosses and coworkers a collaborative versus hostile work environment, and
- whether you are compensated appropriately financially for your work.

[Open-ended response]

1. My own health. Your description could include:

- any chronic health conditions (diabetes, asthma, COPD, autoimmune conditions, etc.),
- frequency of recent colds/flu,
- whether you were infected with COVID19,
- whether you are able to exercise and eat well,
- ability to access needed healthcare,
- whether/how much you use nicotine products, and
- whether/how much you use alcohol.

[Open-ended response]

1. The health of my immediate family members (or does not apply if no immediate family). Your description could include:

- your immediate family’s chronic health conditions (diabetes, asthma, COPD, autoimmune conditions, etc.),
- their frequency of recent colds/flu,
- whether they were infected with COVID19,
- whether they are able to exercise and eat well,
- their ability to access needed healthcare,
- whether/how much they use nicotine products, and
- whether/how much they use alcohol.

[Open-ended response]
