# Supplementary material for: Researcher-Rated “Snapshots” of Stress: Initial Validation of Two Stress Assessment Approaches and Their Relationship to Internalizing Symptoms
Source: Depress Anxiety. 2025 Oct 30;2025:5522234. doi: 10.1155/da/5522234 (PMC12591814; doi:10.1155/da/5522234)
Supplement: Supporting Information 2 — TREND (Transparent Reporting of Evaluations with Nonrandomized Designs) Checklist. In accordance with Depression and Anxiety's policies and in support of data transparency and accountability, we have provided a document describing how our study meets criteria for the TREND checklist. [file 5522234.f2.docx]

| **Section** | **Item** | **Description** | **Completed (Yes/No/NA)** | **Notes** |
| --- | --- | --- | --- | --- |
| **Title and Abstract** | 1 | Title and abstract should indicate the study design, target population, intervention, and outcomes | Yes | No intervention was used, but study design, participants and outcomes were described. |
| **Introduction** | 2 | Scientific background and rationale for the intervention being tested | Yes | Yes a full literature review was provided to explain the research question and hypotheses. |
|  | 3 | Specific objectives and hypotheses | Yes | Where applicable specific hypotheses were provided. |
| **Methods – Participants** | 4 | Eligibility criteria for participants | Yes | Eligibility criteria and notes on exclusions was provided. |
|  | 5 | Recruitment methods and settings | Yes | Sampling method and survey program used provided. |
|  | 6 | Method of assignment to study condition | N/A | There were no separate conditions. |
| **Methods – Interventions** | 7 | Detailed description of the intervention, including content, delivery method, duration, and adherence | N/A | The content of questionnaires was provided. No intervention was administered. |
| **Methods – Objectives** | 8 | Clearly defined primary and secondary outcome measures | N/A |  |
| **Methods – Sample Size** | 9 | How sample size was determined, including calculations and assumptions | Yes | Provided information on inclusions/exclusions. |
| **Methods – Analysis** | 10 | Statistical methods used to compare groups for primary outcomes, and methods for additional analyses (e.g., subgroup, sensitivity) | N/A |  |
|  | 11 | Methods for handling missing data | Yes |  |
|  | 12 | Description of any interim analyses and stopping rules | N/A |  |
| **Results – Participant Flow** | 13 | Flow of participants through each stage (enrollment, allocation, follow-up, analysis); ideally a flow diagram | N/A | Participants were recruited and completed the survey (no conditions/intervention). |
| **Results – Recruitment** | 14 | Dates defining recruitment and follow-up periods | Yes | Recruitment start and end dates provided. |
| **Results – Baseline Data** | 15 | Baseline demographic and clinical characteristics of participants for each group | Yes |  |
| **Results – Numbers Analyzed** | 16 | Number of participants included in each analysis, by group | N/A | No group assignment. |
| **Results – Outcomes** | 17 | Outcomes and estimation (effect size, confidence intervals) | Yes | Standardized betas, p-values, etc. reported. |
| **Results – Ancillary Analyses** | 18 | Results of subgroup or other additional analyses | N/A | All analyses are reported. |
| **Results – Adverse Events** | 19 | Summary of all important adverse events or unintended effects | N/A | Low risk study with low possibility for risk and unintended effects. |
| **Discussion – Interpretation** | 20 | Interpretation of results, considering study hypotheses, sources of potential bias, and limitations | Yes | Results are described in simple language and interpreted in relation to current research. A limitations section is provided. |
| **Discussion – Generalizability** | 21 | External validity: how results can be applied to other populations/settings | Yes | We acknowledge the homogeneity of the sample and discuss a need to extend to other populations. |
| **Discussion – Overall Evidence** | 22 | Overall interpretation of results in the context of current evidence | Yes | Yes, we link the results to current research and identify areas for future direction. |
